# Stem Cell Metabolism: Powering Cell-Based Therapeutics

**DOI:** 10.3390/cells9112490

**Published:** 2020-11-16

**Authors:** Vagner O. C. Rigaud, Robert Hoy, Sadia Mohsin, Mohsin Khan

**Affiliations:** 1 Center for Metabolic Disease Research (CMDR), Lewis Katz School of Medicine, Temple University, Philadelphia, PA 19140, USA; Vagner.rigaud@temple.edu (V.O.C.R.); Robert.hoy@temple.edu (R.H.); 2 Cardiovascular Research Center (CVRC), Lewis Katz School of Medicine, Temple University, Philadelphia, PA 19140, USA; Sadia.mohsin@temple.edu; 3 Department of Physiology, Lewis Katz School of Medicine, Temple University, Philadelphia, PA 19140, USA

**Keywords:** cell therapy, myocardial injury, stem cells, metabolism, metabolic reprogramming

## Abstract

Cell-based therapeutics for cardiac repair have been extensively used during the last decade. Preclinical studies have demonstrated the effectiveness of adoptively transferred stem cells for enhancement of cardiac function. Nevertheless, several cell-based clinical trials have provided largely underwhelming outcomes. A major limitation is the lack of survival in the harsh cardiac milieu as only less than 1% donated cells survive. Recent efforts have focused on enhancing cell-based therapeutics and understanding the biology of stem cells and their response to environmental changes. Stem cell metabolism has recently emerged as a critical determinant of cellular processes and is uniquely adapted to support proliferation, stemness, and commitment. Metabolic signaling pathways are remarkably sensitive to different environmental signals with a profound effect on cell survival after adoptive transfer. Stem cells mainly generate energy through glycolysis while maintaining low oxidative phosphorylation (OxPhos), providing metabolites for biosynthesis of macromolecules. During commitment, there is a shift in cellular metabolism, which alters cell function. Reprogramming stem cell metabolism may represent an attractive strategy to enhance stem cell therapy for cardiac repair. This review summarizes the current literature on how metabolism drives stem cell function and how this knowledge can be applied to improve cell-based therapeutics for cardiac repair.

## 1. Introduction

Cardiovascular disease remains the leading cause of mortality and morbidity in the United States and worldwide [1]. The adult human heart possesses a limited cardiomyocyte turnover rate, which is therefore unable to respond to the massive cellular loss that underlies the development of heart failure [2]. Cell-based therapies have been developed as an alternative strategy to support endogenous generation of new and functional cardiomyocytes. To date, different sources of cells such as bone-marrow derived cells, cardiac progenitor cells, embryonic and inducible-pluripotent stem cell-derived cardiomyocytes have been evaluated in order to identify the most appropriate cell type for cardiac repair. These cell types have been shown to confer therapeutic benefits to the injured heart and affect endogenous cardiomyocyte renewal or directly generate new cardiomyocytes [3]. However, the degree of new cardiomyocyte formation depends on the survival and retention of various stem cells in the heart [3]. Several preclinical cell-based studies have shown remarkable efficacy for cardiac repair but a similar effect was not observed in human clinical trials [4]. This lack of success has been mainly attributed to the poor survival and minimal integration of the transplanted cells into the host tissue as well as lack of an understanding of the mechanisms at play that regulate the benefits associated with cell-based therapeutics [3], which merits the need for understanding the biological properties of stem cells.

Cell-based therapies typically rely on ex vivo expansion of cells under ambient oxygen and high glucose levels that favor mitochondrial respiration for energy generation [5]. Upon transplantation, donated stem cells are exposed to ischemic cardiac milieu composed of inflammatory cytokines and reactive oxygen species (ROS), which alter metabolism. While the metabolic shift towards oxidative phosphorylation (OxPhos) may stimulate the cells to differentiate, it may also result in increased release of ROS, a byproduct of mitochondrial activity that subsequently leads to cellular damage and senescence [5]. Noteworthy, energy metabolism has been reported as a key factor in essential stem cell processes involving proliferation, self-renewal, and cell fate. Understanding stem cell metabolism may define metabolic features of stem cells during ex vivo expansion, proliferation, and post-transplantation survival with potential to enhance cell-based therapies. In this review, we discuss different strategies targeting stem cell metabolism and their potential application to enhance cell survival and therapeutic efficacy for cardiac repair.

## 2. Metabolism at the Heart of Stem Cells

Energy metabolism consists of oxidative processes through which cells generate energy to fuel biochemical processes. Alterations in the microenvironment push stem cells to adopt distinct metabolic states to generate energy by varying substrate utilizations and mitochondrial activities (Table 1). Once viewed as a mere consequence of a specific cell state, recent studies have suggested that metabolism is a finely regulated process that plays a critical role in dictating stem cell fate and adaptation to their microenvironments. The following sections take an in-depth look at metabolic adaptations in stem cells and consequences for the function of adult stem cells.

### 2.1. Quiescence

**Figure 1:**
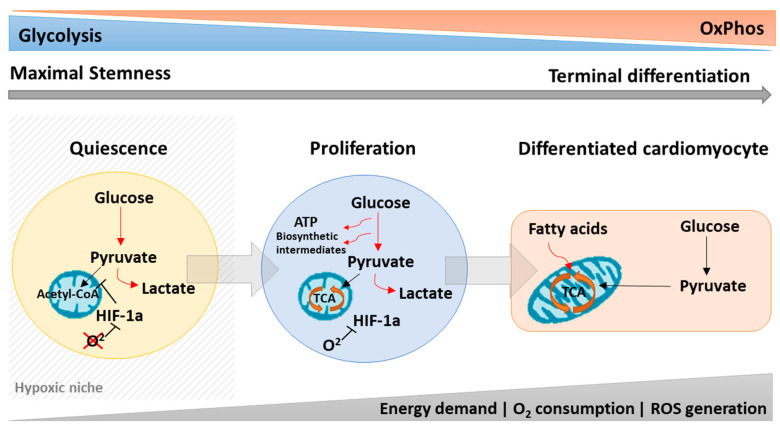
Stem cell metabolism is dynamically modulated to control stemness, proliferation, and cell commitment. Quiescent stem cells are mostly glycolytic due to HIF-1α activity in the hypoxic niche with low generation of ROS to maintain stemness. Outside the hypoxic niche, the oxygen levels begin to rise increasing the oxidative phosphorylation (OxPhos) and reactive oxygen species (ROS) levels, which stimulate the cells to proliferate and differentiate. During proliferation, stem cells mainly rely on glycolysis while still maintaining low OxPhos levels to fuel the cells with biosynthetic intermediates important for cell growth. Stem cell differentiation to cardiomyocytes, however, depends on a metabolic shift from glycolysis to OxPhos in a ROS-dependent manner.

Adult stem cells are tissue resident stem cells thought to support tissue homeostasis and cellular turnover following injury [23] (Wagers and Weissman 2004) [23]. In uninjured tissues, these cells live in a “latent” cell cycle-arrested state known as quiescence. Although quiescent cells do not divide, they retain the ability to re-enter the cell cycle and proliferate in response to environmental stimuli [6,24]. Most of the adult stem cells, including hematopoietic stem cells (HSCs) and cardiac stem/progenitor cells, are found to reside in hypoxic niches in a quiescent or slow-cycling stage [7,25,26,27]. This low-oxygen microenvironment is not only tolerated by these cells, but also seems to be essential for their function. While poorly understood, survival in hypoxic niches requires significant metabolic adaptations with the quiescent stem cell mainly operating under glycolysis that shifts to OXPHOS once cells proliferate or commit towards cardiac lineages (Figure 1).

Within the heart, the epicardium and sub-epicardium have been identified as a cardiac hypoxic niche housing hypoxia-resistant progenitor population, which preferentially utilize glycolytic metabolism for energy production [28]. Indeed, in microenvironments with low-oxygen tensions, adult stem cells cannot rely on electron transport chain (ETC) activity to generate energy and rather prefer anaerobic glycolytic metabolism [6,29]. Hypoxic c-kit+ cardiac stem cells and HSCs are known to reduce mitochondria, mitochondrial membrane potential, oxygen consumption rate, and intracellular ATP levels [6,7,8]. This metabolic state seems to be accompanied by changes in the expression of key metabolic genes such as GLUT1, lactate dehydrogenase α (LDHA), and PDK1, contributing to the increased uptake of glucose and disposal of pyruvate [29,30]. Recent studies have shown HIF-1α as a master regulator of both glycolysis and mitochondrial respiration [6,28,31,32]. Under hypoxic conditions, HIF-1α is stabilized and induces the expression of PDK2 and PDK4, which in turn prevent the conversion of pyruvate into acetyl-CoA by inhibiting PDH, and thus hampering mitochondrial respiration [6,28,31,32].

Noteworthy, a reliance on anaerobic glycolysis and reduction in OxPhos is also believed to be, at least in parts, necessary to maintain quiescence by protecting c-kit+ cardiac progenitor cells and HSCs from ROS, a known contributor to ageing and senescence [33,34,35].

### 2.2. Proliferation

Quiescent adult stem cells are reversibly arrested in G0 to maintain their stemness [5]. However, following stimulation or injury, quiescent cells quickly re-enter the cell cycle, proliferate, and commit into specific tissue lineages to replace damaged cells [29]. The transition from a quiescent to a lineage-committed state is characterized by migration from a hypoxic niche to an oxygen-rich microenvironment [5]. In the presence of oxygen, mitochondrial activity increases, which generates ROS, believed to induce adult stem cell proliferation but also apoptosis at elevated concentrations [24,29]. Thus, energy metabolism in proliferating cells markedly differs from that in quiescent cells.

With higher oxygen tension, HIF-1 levels decrease through oxygen-mediated ubiquitination and proteasomal degradation, which affects proliferation [5]. Levels of HIF-1α target genes, such as PDK2 and PDK4, decrease, dephosphorylating PDH and leading to the oxidation of pyruvate into acetyl-CoA [5]. Increased acetyl-CoA feeds the TCA cycle, increasing mitochondrial respiration and thereby switching the metabolism from anaerobic glycolysis to OxPhos. Indeed, mesenchymal stem cells (MSCs) cultured under normoxia have been shown to rely on OxPhos and high oxygen consumption [36]. Additionally, these cells present an increased proliferation rate [37]. However, the metabolic switch to OxPhos is associated with higher mitochondrial activity, which generates ROS and over a long time leads to a significant increase in senescence, which impairs MSC stemness [37].

Studies have shown that the transition from quiescence to a more proliferative state in HSCs has been shown to be driven by two different pathways, TSC-mTOR [38] and the mitochondrial carrier homolog 2 (MTCH2) [39]. Disruption in one of these pathways leads to increased mitochondrial biogenesis and size, ATP production and ROS, which drive HSCs from quiescence to a rapidly cycling state that reduces stemness. Unlike HSCs, c-Kit+ cardiac progenitor cells (CPCs) express high levels of GLUT1 transporter in an undifferentiated state, a feature also seen in embryonic stem cells [17,18,40], while expressing a glutamine transporter (ASCT2) similar to HSCs, with their mitochondrial activity responsive to glutamine (discussed later in the substrate utilization section) [17]. These findings suggest that, while sharing metabolic characteristics with both embryonic stem cells and HSCs, CPCs present a unique metabolic phenotype different from other stem cells. Although increasing extracellular glucose concentration elevates glycolytic rate in CPCs, only a marginal or negative effect on proliferative states is observed concurrent with the increase in apoptosis [17,41]. This harmful effect of high glucose levels might be associated with an impairment in mitochondrial respiration via a Crabtree-like effect—a phenomenon particularly observed in proliferating cells where an increase in glucose concentrations accelerates glycolysis, reducing the need of OxPhos [17,18]. Thus, similar to HSCs and mesenchymal stem cells (MSCs), CPCs apparently do not depend on glycolysis to proliferate under normoxia. Glutamine-mediated mitochondrial activity, however, has been shown to be essential for both c-kit+ and Sca1+ CPC proliferation and growth suggesting that the stimulatory effect of glutamine is likely due to anaplerosis [17].

Recently, Cited2, a protein implicated in glucose metabolism, has been shown to be important for Sca-1+ cardiac stem cell proliferation [42]. Although the authors did not measure any metabolic parameter, Cited2 deletion in ESCs has been shown to result in aberrant mitochondrial morphology, reduced glucose oxidation, increased glycolysis, and defective differentiation [43]. Our recent work has identified a novel role for uncoupling protein 2 (UCP2) in maintaining cardiac tissue-derived stem cell (CTSC) proliferation during the transition from young to old age [44]. CTSCs from neonatal hearts have elevated UCP2 together with increased glycolysis and reduced mitochondrial respiration, which promotes rapid proliferation rates. With age, UCP2 is lost, promoting a shift towards OxPhos from glycolysis and adversely affecting proliferation [44].

Taken together, the transition from quiescent cardiac stem cells to a more proliferative state seems to be associated with their migration to an oxygen-rich environment and a metabolic switch from anaerobic glycolysis to a glutamine-mediated oxidative metabolism with activation of a mitochondrial gene program.

### 2.3. Survival

The harsh microenvironment within the injured host myocardium is one of the major obstacles for cell-based therapies. Low oxygen tension, high oxidative stress, inflammatory response, and deprivation in nutrient supply together create a barrier for the successful engraftment and survival of the transplanted stem cells.

As previously discussed, when cultured under normoxia, cardiac stem cells adapt their metabolism shifting towards oxygen dependent OxPhos, which inadvertently compromises cell survival following transplantation into a hypoxic/ischemic environment typical of cardiac injury. This “metabolic shock” can be attenuated by activating glycolytic pathways through preconditioning cardiac stem cells in hypoxia prior transplantation, which has indeed been shown to improve survival in the myocardium [45,46,47], although the metabolic adaptations were not addressed in these studies. Similarly, preconditioning of cardiosphere-derived cells (CDCs) in hypoxia has been shown to induce glucose uptake, lactate production and decrease cellular oxygen respiration, suggesting metabolic adaptation towards glycolysis and reduced dependence on OxPhos [48]. Mechanistically, metabolic changes were linked to stabilization of HIF1-α even when oxygen was present. HIF1-α stabilization led to the expression of gene targets that inhibit the formation of acetyl-CoA and the subsequent TCA cycle activity, forcing the cells to rely mostly on glycolysis and preserve stemness. In CPCs, increased extracellular glucose concentrations lead to diminished proliferation, lower survival [40], and reduced ability to repair the injured myocardium [49], suggesting that CPCs are susceptible to hyperglycemic injury [17]. Interestingly, the increase in apoptosis in both CPCs cultured in high glucose media or isolated from diabetic hearts seems to be associated with a deficiency in the pentose phosphate pathway (G6PD) [17,41]. Reactivation of the G6PD pathway in vivo and in vitro by supplementation with benfotiamine rescues CPC survival and function, an effect that is abrogated by G6PD silencing by siRNA [41]. Thus, G6PD seems to play a crucial role in CPC survival under diabetic conditions, a feature that has also been also reported in cardiomyocytes [50]. Our recent work regarding the RNA-sequencing approach demonstrated survival signaling pathways to be significantly altered in cardiac-derived stem like cells (CTSC) after loss of mitochondrial uncoupling protein 2 (UCP2) together with decreased glycolysis, thereby identifying a novel role for UCP2 in CTSC survival [44].

Similarly, mimicking hypoxia by either preconditioning MSCs with prolyl hydroxylase inhibitors [51] or shRNA knock down [52] leads to increased survival and angiogenesis following transplantation into ischemic hearts. Furthermore, hypoxia-induced HIF-1α upregulation was also shown to stimulate glucose uptake and upregulate expression of glycolytic genes, which promotes MSC survival in response to long-term ischemia and enhances therapeutic efficacy [53]. Taken together, HIF-1α upregulation either by hypoxia or prolyl hydroxylase inhibition improves adult stem cell survival and therapeutic efficiency, at least in parts, by promoting metabolic adaption to low oxygen environments such as ischemic myocardium [31]. However, to maintain anaerobic metabolism for long-term survival in hypoxic conditions, a persistent glucose supply is required. The use of glucose-loaded scaffolds has been shown to lead to a five-fold enhancement of MSC survival rate post implantation together with increased HIF-1α expression [54]. Although anaerobic glycolysis is critical for cells to generate ATP under ischemia, MSC survival seems to be dependent on glycolysis rather than OxPhos, not only in hypoxia, but also in normoxia. In line with the previous discussion, disruption of the ETC by either antimycin A or malanate under low oxygen levels [55] or inhibition of OxPhos under standard culture conditions does not impair MSC metabolism nor increase the cell mortality rate up to 72 h post treatment [56]. However, inhibition of glycolysis resulted in a significant decrease in cell viability as early as 24 h after treatment [55,56], suggesting only glycolysis is essential for MSC survival.

### 2.4. Stem Cell Commitment

Egress from the hypoxic niche is accompanied by stem cell proliferation and commitment or apoptosis, dependent on the local stimuli. Although there is a large body of evidence regarding metabolic transitions underlying ESC and induced pluripotent stem cell (iPSC) differentiation into cardiomyocytes, not much is known about adult stem cells. The energetic requirements of proliferating adult stem cells and mature cardiomyocytes are believed to be different since cardiomyocytes no longer need to sustain high proliferation rates and therefore have lower anabolic demands. However, the lower requirement for anabolic precursors enables cardiomyocytes to catabolize substrates in a more energy efficient manner within the TCA cycle, yielding large amounts of energy to fuel sustained contraction.

As discussed previously, increase in oxygen tension is a key process regulating CPC proliferation and lineage commitment through degradation of HIF-α, which subsequently allows pyruvate to be converted into acetyl-CoA to fuel the TCA cycle. In CPCs, downregulation of HIF-1α leads to a metabolic shift from glycolysis to mitochondrial OxPhos, resulting in loss of the uncommitted state [28]. Interestingly, this HIF-α-induced commitment seems to favor cardiomyocyte and endothelial lineage commitment over smooth muscle or fibroblast generation [28]. Similarly, Sca1+ CPCs undergo metabolic shift towards OxPhos together with a reduction in glucose consumption, lactate release, and glycolysis related genes such as GLUT1, MCT4, and PFK2, and an increase in mitochondrial content, and respiration rate [18].

Since mitochondria mass represents up to 40% of the volume of a mature cardiomyocyte [57], mitochondrial biogenesis is undoubtedly a crucial process during differentiation and defects in mitochondria have been shown to impair CPC commitment leading to cell death [58]. Mitochondrial biogenesis-related gene PGC-1α is upregulated during CPC commitment [18], and its activation by resveratrol [59] or oleic acids [19] has been shown to improve commitment and enhance reparative properties of CPCs following acute myocardial infarction. Alternatively, increased mitochondrial biogenesis was not observed in Sca1+ CPCs after pharmacological activation of the AMPK/PGC-1α pathway during commitment [18]. In MSC, AMPK activation led to an increase in the expression of mitochondrial biogenesis markers; however, it was not sufficient to drive their metabolism to OxPhos [60]. Taken together, these results suggest that, although essential, mitochondrial biogenesis may not be sufficient to trigger adult stem cell commitment, suggesting a role for additional molecular processes.

Recently, mitochondrial autophagy (or simply mitophagy), an important process for mitochondrial reorganization, has also been implicated in CPC commitment [61]. Mitophagy is rapidly induced upon initiation of cardiac commitment and seems to be required for the formation of a functional interconnected mitochondrial network by eliminating impaired and immature mitochondria [61]. Furthermore, impairment of mitophagy resulted in mitochondrial fission, increased susceptibility to oxidative stress-mediated cell death, and reduced CPC retention in vivo [61].

### 2.5. Substrate Utilization

The adult heart is known as a metabolic “omnivore” able to utilize a wide range of substrates for energy production. Factors including nutrient availability, developmental/disease stage, and cell-specific preferences influence substrate choice, which may impact cellular function. For example, PSCs utilize mostly glucose while fully differentiated CMs generate 70% of their ATP through fatty acid (FA) oxidation [62]. This shift in substrate utilization seems to be essential for CM differentiation, and maturation, and is also believed to play a role in CM cell cycle arrest [63].

Cardiac progenitor cells are primarily dependent on glutamine [17,18]. After treatment with different substrates, Salabei and colleagues [17] showed that stimulation of Sca1+ CPCs with glutamine was the most effective to support mitochondrial respiration, cell proliferation and viability. The effects were reversed by treating the cells with rapamycin, leading to an 80% reduction in glutamine-induced proliferation [17]. The beneficial effects of glutamine utilization on CPCs are related to the generation of byproducts of glutamine oxidation. Supplementation with cell permeable α-KG was sufficient to stimulate both mitochondrial activity and cell proliferation, while inhibiting glutaminolysis led to a significant decrease in CPC proliferation [17]. A recent study has reported that both glutamine and glucose together are necessary for the growth and proliferation of Sca1+ CPCs [18]. Similar to CPCs, many other proliferating cells, such as MSCs [64], utilize glutamine as a source for biosynthesis. Rather than being a substitute fuel for energy generation, glutamine can supply the TCA cycle with intermediates that can be used to synthesize new macromolecules that support cell growth [65]. Taken together, these findings suggest that interventions to increase glutamine availability, uptake or consumption might increase the proliferative and survival capability of stem cells and potentially impact cardiac repair after transplantation.

Although the influence of fatty acid utilization in PSC and CM metabolism and function is well-documented, little is known about their role in adult stem cells. This is of critical importance since adult stem cells are usually expanded in a culture medium containing high levels of glucose and no fatty acids, whereas after transplantation, plasma levels of glucose are generally low and enriched in fatty acids. This difference in substrate availability may cause the cells to experience a metabolic shift and potentially impact their reparative capacities. In this regard, Malandraki-Miller and colleagues [19] showed that the addition of oleic acid into the culture medium enhanced the maturation of the Sca1+ CPCs by stimulating the PPARα-fatty acid oxidation pathway. Furthermore, the authors observed an increase in glycolytic metabolism, mitochondrial membrane potential, and glucose and fatty acid oxidation [19]. In MSC, treatment with physiological levels of different fatty acids revealed that oleic acid does not impact on cell viability; however, it does protect the cells against palmitate-induced apoptosis and decreased proliferation [21]. Interestingly, palmitate-induced apoptosis seems to be associated with a reduction in fatty acid oxidation rates, which was prevented by the addition of oleic acid [21]. Since MSCs primarily rely on glycolysis for ATP production (97%) [21], the impact of reduced fatty acid oxidation on proliferation and survival is unlikely to be related to a depletion in energy production, which suggests an alternate mechanism.

These studies demonstrate how substrate utilization can drive phenotypic changes in stem and progenitor cells, which may lead to effective strategies to enhance stem cell therapy for cardiac repair.

### 2.6. Redox Homeostasis

Reactive oxygen species (ROS) are mainly generated because of electron leak from the electron transport chain during mitochondrial oxidative metabolism. ROS have been known to promote widespread damage in several cellular components such as DNA, proteins, and lipids. Although initially recognized as harmful, new evidence suggests that ROS are key regulators of stem cell biology. This double-edged role seems to be dependent on the amount of ROS that are produced. High levels are associated with stem cell senescence, premature exhaustion, and apoptotic death while low levels can modulate the balance between self-renewal and lineage commitment to cardiomyocytes [9,66,67]. Therefore, understanding the fine balance required for maintenance of intracellular ROS and regulation of adult stem cell function potentially holds significance for cardiac reparative processes [68].

As previously discussed, adult cardiac stem cells are maintained in a quiescent state within a hypoxic niche, which helps shield them from oxidative stress. In HSC, Meis1 appears to play a critical role in this process towards transcriptional activation of HIF-1α and HIF-2α, and thus activating cytoplasmic glycolysis, and antioxidant defense mechanisms to suppress ROS production [29]. Furthermore, treatment with ROS scavengers seem to revert cells back to quiescence [69], suggesting hypoxic niche favors quiescence via activation of HIF-1-mediated metabolic shift, which reduces mitochondrial OxPhos, the main source of ROS, and enhances anaerobic glycolysis.

As a byproduct of ETC utilization, the increase in ROS production is critical for stem cell commitment. In Sca1+ CPCs, ROS were directly linked with cardiomyocyte-related gene expression in vivo while antioxidant treatment partially blocked ROS-mediated commitment. Furthermore, authors suggested that ROS act, at least in part, through epigenetic modifications related to the polycomb repressive complex component (BMI1), an important player in DNA repair and redox regulation [9]. BMI1 was shown to repress a cardiogenic differentiation program. However, an increase in intracellular ROS modifies BMI1 activity, suppressing target genes related to cell fate decisions [9], implying ROS levels as key for the balance between stemness and cell fate.

Preconditioning of MSCs with 1-[2,3,4-trimethoxybenzyl]piperazine (TMZ), a drug used to reduce tissue demand for oxygen, resulted in significant protection against H2O2-induced stress, membrane damage, and oxygen metabolism. Following adoptive transfer, TMZ-preconditioned MSCs resulted in an improved heart function and decreased myocardial fibrosis [70]. Although the glycolytic flux was not assessed in this study, TMZ is believed to act by stimulating anaerobic glucose oxidation in the ischemic myocardium [71].

Although mitochondria are the main sources of ROS in most cells, NADPH oxidase enzymes (Nox) are another major contributor. Nox is believed to be an essential component of the cardiac redox system by generating the necessary amount of ROS to drive cardiomyogenesis. During c-kit+ CPCs commitment, Nox2 and Nox4 levels are low under basal conditions but increase over the course of commitment, parallel with ROS levels [72]. Interestingly, knocking down Nox4 in either c-kit+ CPCs [72] or ES cells [73,74] was shown to reduce generation of beating cardiomyocytes while exposure to low levels of ROS by exogenous H2O2 administration seems to revert it, enhancing commitment [73]. Moreover, NADPH oxidase seems to be involved in a feed-forward regulation of ROS generation since protein levels increase when the cells are treated with ROS [66,74,75].

In addition to changes in ROS production, alterations in the antioxidant balance may also be involved in regulating cardiomyocyte commitment. Comparing freshly isolated c-kit+ CPCs with cardiomyocytes, a set of 27 redox genes were divergent, including upregulation of the superoxide dismutase (Sod1, Sod2) and peroxiredoxin (Prdx2, Prdx3, Prdx5, Prdx6) families, and downregulation of pro-oxidant p67^phox^ [72]. A separate study identified APE1/Ref-1—a protein involved in DNA repair and redox balance—maintaining redox status and survival of cardiac stem cells [66]. Furthermore, Sca1+ CPCs overexpressing APE1/Ref-1 were resistant to oxidative stress, reduced fibrosis and enhanced cardiac repair 28 days after myocardial infarction [76]. In line, CSCs pre-conditioned with resveratrol, a natural antioxidant present in red wine, were also shown to increase APE1/Ref-1 expression. Transplantation of these cells leads to enhanced cell survival and engraftment, and ultimately improved cardiac function up to 4 months after transplantation [77].

## 3. Metabolism and Cellular Reprogramming

**Figure 2:**
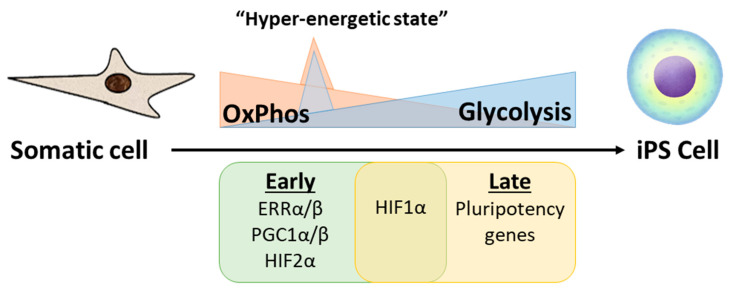
Somatic cell reprogramming to induced pluripotent stem cell (iPSC) involves a metabolic shift from OxPhos to glycolysis. The early expression of ERRα, ERRβ, and PGC1α/β precedes a metabolic burst leading the cells to a hyper-energetic state, which is followed by a progressive increase in glycolysis and decrease in OxPhos.

The reprogramming of a fully differentiated somatic cell into iPSC involves profound changes in several cellular processes including the transcriptome, epigenome, morphological, and functional programs. In addition, reprogramming requires major metabolic changes to meet the different energetic and functional requirements of the iPSC (Figure 2). As discussed previously, increased glycolytic flux of PSCs is important to maintain stemness, while upregulation of OxPhos is necessary for cell differentiation. Thus, the general trend during reprogramming involves shifting the metabolism back to a more “primitive” state by decreasing OxPhos and increasing glycolysis [78,79,80].

During the early phase of reprogramming, the increase in glycolysis is accompanied by a transient burst in OxPhos activity to promote a temporary hyperenergetic state induced by transient expression of ERRα, ERRβ, and *PGC1α/β* [81]. These findings agree with a previous study showing transient elevation of mitochondrial proteins in cells undergoing reprogramming, and a progressive increase in glycolysis [82]. One possible mechanism to explain an oxidative burst in the early stages is through the increase in one of the OxPhos byproducts, ROS. In this respect, Hawkins and colleagues [83] showed early OxPhos burst increases ROS levels leading to an activation of HIF1-α and subsequently promoting a glycolytic shift and glucose redistribution to the pentose phosphate pathway in a process, at least in part, controlled by KEAP1 and NRF2. Additionally, HIF1-α and HIF2-α activation has been reported to be required for human fibroblast reprogramming into iPSC [84]. This metabolic shift seems to be crucial for acquisition of pluripotency since it occurs in the early stages and precedes expression of pluripotent genes [78,84,85]. At least in part, this metabolic shift may be induced by some key reprogramming factors such as c-Myc and LIN28, which are known regulators of energy metabolism and have been shown to enhance glycolysis [86,87,88] and suppress OxPhos [88]. Moreover, the function of c-Myc in inducing pluripotency can be replaced by the overexpression of enzymes involved in glycolysis such as LDHA and PKM2, suggesting one of the main roles of the reprogramming factors is to enhance glycolysis [86]. In the latter stages of reprogramming, the oocyte factors Tcl1 and Tcl1b1 play an additional role in supporting the metabolic shift and their upregulation enhances reprogramming efficiency [89,90]. Mechanistically, Tcl1 increases Akt1 activity, further increasing expression of glycolytic enzymes, while Tcl1b1 inhibits OxPhos and mitochondrial biogenesis by suppressing mitochondrial localization of the polynucleotide phosphorylase (PnPase). Thus, contributing to the switch from oxidative metabolism to glycolysis during reprogramming [89].

Mitochondria biology that regulates cellular metabolism also has a fundamental role in reprogramming. Along with a reduction in OxPhos, mitochondrial mass and enzymes involved in the ETC gradually decrease during the course of reprogramming [82,91]. Morphologically, mitochondria shift back to a more ESC-like phenotype, altering from an elongated tubular shape with well-developed cristae to a smaller sized and spherical form with poor-developed cristae [92]. In addition, mitochondria cellular distribution changes from a complex mitochondria network distributed within the cytoplasm to a primarily peri-nuclear localization [92]. The mechanisms underlying this mitochondria remodeling remain unclear and appear contradictory in some ways. Mitophagy, for example, has been shown to play an important role by selectively clearing mature mitochondria as new immature mitochondria are produced [91,93,94]. The mitophagy process is governed in either an Atg-dependent manner through the repression of mTOR [93] or in an Atg-independent manner through the activation of AMPK [91]. On the other hand, the decrease in mitochondria size has been credited to mitochondria fragmentation, which was attributed to mitochondrial fission through the expression of pro-fission dynamin-related protein 1 (DRP1), induced by ERK1/2 in early reprogramming [95]. In a more recent study from the same authors, c-Myc was shown to indirectly induce phosphorylation of DRP1, resulting in mitochondrial fission and the hybrid energetic state seen in the early stage of induction of pluripotency [96].

Taken together, energy metabolism is emerging as more than a mere consequence, but a critical driver in somatic cell reprogramming into iPSC. In addition, a metabolic shift from OxPhos to glycolysis is crucial not only in maintaining stemness (as previously discussed), but it is also essential in the acquisition of pluripotency and may have profound implications for iPS-based therapeutics for cardiac repair.

## 4. Metabolic Reprogramming as a Strategy for Cardiac Repair

**Figure 3:**
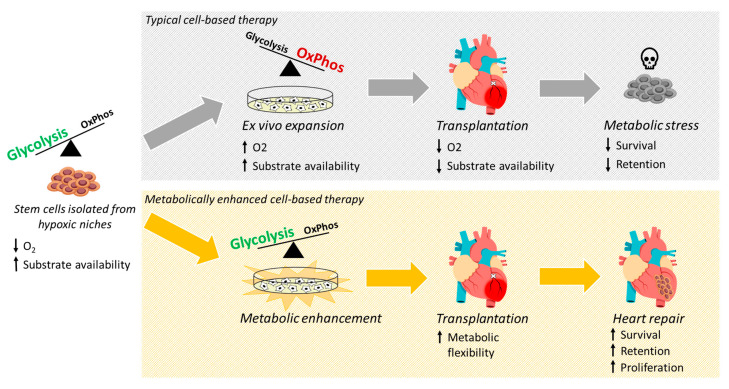
Cell-based therapies typically rely on isolating highly glycolytic stem cells from hypoxic niches followed by ex vivo expansion under high oxygen and glucose levels, which shifts energy generation towards a more oxidative metabolism. Upon transplantation, however, donated stem cells are exposed to an ischemic cardiac milieu characterized by an intense deprivation in oxygen and glucose essential for mitochondrial activity. This rough transition from in vitro to in vivo leads the cells to experience a metabolic stress driving transplanted cells towards apoptosis. Thus, the enhancement of stem cell metabolism holds a great promise towards promoting metabolic flexibility and improving cell survival and therapeutic efficacy.

The role of metabolism in regulating the function of stem cells has opened new avenues to enhance the effectiveness of cell therapy for cardiac repair. Strategies to mitigate the metabolic shock experienced by stem cells after transplantation, as well as to enhance their adaptation to a new ischemic environment, can potentially alleviate cell death and improve success of the therapy (Figure 3).

One of the first studies to modify stem cell metabolism to improve cardiac function after injury was published in 2003 by Mangi and colleagues [10]. In this study, the authors transplanted genetically engineered MSCs overexpressing Akt into an ischemic myocardium resulting in improved cardiac function and decreased inflammation, fibrosis, and hypertrophy, which was associated with a higher MSC survival and retention [10]. Akt is known to regulate glycolysis in stem cells [66,89,97] and MSCs overexpressing Akt have also been shown to have increased glucose metabolism [98]. Thus, overexpressing Akt may push the cells to exhibit an increased glycolytic state, enhancing survival in the ischemic myocardium.

Downstream Akt, Pim-1 kinases have been reported to regulate energy metabolism and cell growth by increasing the protein levels of both c-Myc and PGC-1α [99]. Interestingly, overexpression of Pim-1 either in c-kit+ bone-marrow derived cells (BMCs) [11], c-kit+ mCPCs [12], or c-kit+ hCPCs isolated from patients with heart failure [13,14] has been shown to enhance cardiac repair in both rodent and porcine models of myocardial infarct. In each case, overexpression of Pim-1 resulted in a significant increase in cell survival and proliferation leading to improved cardiac function and structure after transplantation into the infarcted heart. Among the possible mechanisms involved in the salutary effect of Pim-1 overexpression, the authors showed a progressive enhancement in metabolic activity [11,12,13].

As previously discussed, stem cells are typically expanded under normoxia and subsequently transplanted into an ischemic tissue leading to a metabolic shock, which negatively impacts cell retention. Preconditioning MSCs in hypoxic conditions prior to transplantation has been shown to improve cell survival and retention [100]. The beneficial effect of the hypoxic treatment was mainly credited to changes in cell metabolism towards a more glycolytic state, and subsequent alteration of several metabolites [100]. Indeed, inhibition of mitochondrial respiration leads to a compensatory increase in glycolytic and colony forming capacities in human HSCs and BMCs, independent of ATP production [101].

However, key considerations must be made regarding the therapeutic approach for patients with underlying metabolic dysfunction. Derlet and colleagues [101] have shown that the glycolytic capacity of bone marrow cells derived from chronic heart failure (CHF) patients is reduced compared to healthy controls, impacting the colony forming ability and proliferation rates. Moreover, ex vivo inhibition of glycolysis further reduced the pro-angiogenic activity of transplanted cells in a hind limb ischemia model in vivo [101]. Similarly, induction of diabetes profoundly impacts functional characteristics of Sca-1+ CPCs and, importantly, cellular metabolism. Diabetic Sca-1+ CPCs exhibited impaired pentose phosphate pathway (PPP), and inhibition of the Akt/Pim-1/Bcl-2 signaling pathway along with reduced abundance and proliferation. Pharmacological restoration of PPP resulted in improved survival and functional ability of the cells, suggesting metabolic reprogramming as a strategy for enhancement of diabetes affected cell function [41].

As an emerging field, only a few studies are currently available, and more research is still needed on how metabolism can be explored to enhance stem cell function and improve therapy for cardiac repair.

## 5. Conclusions

Beyond fueling cells with ATP, energy metabolism has emerged as a key process in supporting stem cell function and commitment, playing an important role in both the acquisition and maintenance of stemness. The balance between glycolytic and oxidative metabolism involves a complex network regulation that connects the nucleus, cytoplasm, and mitochondria and provides a wide arsenal of molecules that can be manipulated to help stem cells survive the ischemic environment. Thus, reprogramming stem cell metabolism represents a new frontier to enhance stem cell therapy for cardiac repair.

## Figures and Tables

**Table 1:** Metabolic adaptations and functional changes of pluripotent and adult stem cells in response to specific environmental stimuli.

Stimulus	Cell Type	Metabolic Change	Functional Change	Signaling Pathway	References
Hypoxia	c-kit+ CPC HSC	↑ glycolysis ↓ oxphos	↑ stemness ↑ self-renewal	HIF-1α/PDK4	[6,7,8]
ROS	Sca1+ CPC	↓ glycolysis ↑ oxphos	↑ differentiation ↓ stemness	BMI1/epigenetic modification	[9]
Akt overexpression	MSC	↑ glycolysis	↑ survival ↓ inflammation	AKT/eNOS signaling	[10]
PIM1 kinase overexpression	c-kit+ BMC c-kit+ mCPC c-kit+ hCPC	↑ glycolysis	↑ survival ↑ proliferation	AKT/eNOS signaling	[11,12,13,14]
B adrenergic stimulation	c-kit+ hCPC	↑ glycolysis	↑ survival ↑ proliferation	AKT/eNOS signaling	[15,16]
Glutamine	Sca1+ CPC	↑ oxphos	↑ survival ↑ proliferation	TCA intermediates	[17,18]
Oleic Acid	Sca1+ CPC PSC MSC	↑ glycolysis ↑ oxphos ↑ FA oxidation	↑ survival ↑ maturation ↓ apoptosis	PPARα-FA oxidation	[19,20,21]
Lactate	PSC	↑ oxphos	↑ differentiation	TCA intermediates	[22]

↑ increasing; ↓ decreasing; Abbreviations: cardiac progenitor cells (CPC), mesenchymal stem cells (MSC), hematopoietic stem cells (HPC), bone-marrow derived cells (BMC), pluripotent stem cells (PSC).
